# Probiotic Cocktail Alleviates Intestinal Inflammation Through Improving Gut Microbiota and Metabolites in Colitis Mice

**DOI:** 10.3389/fcimb.2022.886061

**Published:** 2022-06-15

**Authors:** Yefei Zhu, Yu Xu, Xinyue Wang, Leiping Rao, Xuebing Yan, Renyuan Gao, Tongyi Shen, Yuan Zhou, Cheng Kong, Longxiang Zhou

**Affiliations:** ^1^Research Institute of Intestinal Diseases, Tongji University, School of Medicine, Shanghai, China; ^2^Department of General Surgery, Jinshan Branch of Shanghai Sixth People’s Hospital, Shanghai University of Medicine and Health Sciences, Shanghai, China; ^3^School of Medicine, Nantong University, Nantong, China; ^4^Department of Oncology, the Affiliated Hospital of Yangzhou University, Yangzhou University, Yangzhou, China; ^5^School of Biological Engineering, Hefei Technology College, Hefei, China

**Keywords:** gut microbiome, gut barrier, short-chain fatty acids (SCFAs), liquid chromatograph mass spectrometer (LC-MS/MS), inflammatory bowel disease (IBD), fecal microbiota transplantation (FMT)

## Abstract

The modulation of the gut microbiome has been widely suggested as a promising therapeutic strategy for inflammatory bowel disease (IBD). Here, we established a novel probiotic cocktail to investigate its therapeutic role in acute colitis mice. During dextran sulfate sodium (DSS)-induced colitis, the mice were treated with the probiotic cocktail, fecal microbiota transplantation (FMT) from a healthy mice donor, or 5-aminosalicylic acid (5-ASA), respectively. The inflammatory responses were assessed by symptoms, serum inflammatory factors, and histological scoring. The intestinal barrier function was assessed by detecting tight junction proteins. Gut microbiota and its metabolites were further identified using 16S rDNA sequencing and a liquid chromatograph mass spectrometer (LC-MS/MS). Compared with FMT and 5-ASA treatment, the probiotic cocktail performed better in alleviating symptoms of colitis and decreasing disease activity score and mucosal inflammation. The probiotic cocktail also significantly decreased serum IL-17 level and increased JAM-1 expression in colon. The gut microbiota analysis confirmed that the beneficial effects of the probiotic cocktail were attributed to increasing anti-inflammatory bacteria *Akkermansia*, *Bifidobacterium*, and *Blautia*, while decreasing pro-inflammatory bacteria *Parasutterella*. The targeted metabolome analysis further indicated a rise in the production of *Bifidobacterium*-related short-chain fatty acids (SCFAs) such as propanoic acid and isobutyric acid after probiotics treatment. Taken together, the probiotic cocktail effectively alleviated intestinal inflammation through improving gut microbiota and metabolites in colitis mice, suggesting its great potential to be a novel therapeutic approach for IBD patients.

## Highlights

1. The probiotic cocktail performed better in alleviating colitis in mice compared with healthy donor FMT and 5-ASA treatment.
2. The probiotic cocktail decreased DAI and mucosal inflammation, and protected the intestinal barrier function in colitis mice.
3. The beneficial effects of the probiotic cocktail were attributed to increasing anti-inflammatory bacteria and *Bifidobacterium*-related short-chain fatty acids in colitis mice.

## Introduction

Inflammatory bowel disease (IBD), characterized by chronic recurrent inflammation involving the whole colon (Wijmenga, 2005), is a major health concern worldwide with respect to the rising incidence in America and Asian countries during the past few decades (Thia et al., 2008; Kerur et al., 2021). Despite the great progress in anti-inflammatory, immunosuppressive (Hernández-Chirlaque et al., 2016), and biological agents, a considerable proportion of IBD patients continue to suffer from recurrence (Loddo and Romano, 2015). Therefore, the development of novel and safe therapeutic strategies for IBD is in critical need.

Emerging evidence suggests that gut microbiota dysbiosis contributes to the pathogenesis of IBD; for instance, opportunistic pathogen derived from ulcerative colitis patients displayed an inflammatory phenotype that caused mice colitis (Lloyd-Price et al., 2019; Seishima et al., 2019). Thus, the emerging methods of manipulating gut microbiota, such as probiotics and fecal bacteria transplantation (FMT), have been advocated as a promising strategy in alleviating intestinal inflammation. Burrello et al. reported that FMT treatment altered intestinal mucosal immunoreactive responses during experimental colitis, primarily by reducing colonic inflammation, increasing colonic barrier function, and simultaneously activating multiple immune-mediated pathways (Burrello et al., 2018). Meanwhile, both animal and clinical studies have demonstrated that the utilization of probiotics could prevent colonic inflammation *via* the regulation of intestinal macrophage differentiation, alternation of various inflammatory cytokines, and the enhancement of gut barrier (Oh et al., 2020; Chen et al., 2021; Dias et al., 2021). The successful colonization of particular probiotics could also prevent pathogenic bacteria, interact with intestinal epithelium, and produce multi-functional beneficial metabolites (e.g., SCFAs and hydroxytryptamine) (Liu et al., 2018; Stojanov et al., 2020). Nonetheless, the probiotics selected and analyzed in most cases were confined into one or several substrains, which cannot draw a consistent and convincing conclusion. Thus, we developed the probiotic cocktail strategy and evaluated the therapeutic potential of bacteria manipulation for IBD.

In this study, the probiotic cocktail contains 3 *Bifidobacterium* and 7 *Lactobacillus* substrains. Although numerous intestinal inflammation models have been developed to investigate IBD (Kiesler et al., 2015), here we utilized the dextran sulfate sodium (DSS)-induced colitis mice model to investigate the probiotic cocktail’s therapeutic effects. DSS is a water-soluble sulfated polysaccharide that has been widely used to construct a colitis murine model (Okayasu et al., 1990) due to its simplicity, efficiency, and repeatability (Chassaing et al., 2014). Meanwhile, considering 5-aminosalicylic acid (5-ASA) being the first-line clinical drug for the treatment of IBD patients (Magro et al., 2020), 5-ASA treatment together with healthy mice donor FMT were employed for comparison. Furthermore, microbial sequencing and targeted metabolomics were used to clarify its beneficial role on gut microbiota and metabolites in mice. Our study identified the colitis alleviation and the characteristics of gut microbiota and metabolites after the probiotic cocktail treatment, providing a novel probiotics-based therapeutic approach for IBD.

## Materials and Methods

### Study Design and Animal Treatment

Twenty-five male C57BL/6 mice (8 weeks old) (Shanghai SLAC Laboratory Animal Co., Ltd.) were purchased and caged under specified pathogen-free (SPF) conditions (Experimental Animal Center, Hubei Campus, Tongji University, Shanghai, China) at 22 ± 2°C with 55 ± 15% humidity and a 12-h dark/12-h light cycle. All the mice had free access to normal diet (Ralston Purina, St. Louis, Missouri, USA). Then, a continuous 7-day colitis induction was performed using 3% DSS (36–50 kDa, Sigma, US, LOT NO: S3045) dissolved in drinking water (Liu et al., 2016). Meanwhile, the mice were randomly assigned to 5 different groups before treatment: the blank group (mice gavaged with PBS only), the control group (mice treated with DSS and gavaged with PBS), the probiotics group (mice treated with DSS and gavaged with probiotic cocktail), the FMT group (mice treated with DSS and gavaged with fecal suspension from healthy mice), and the ASA group [mice treated with DSS and gavaged with 5-ASA (100 mg/kg) (JiaxingSiCheng Chemical Co., Ltd., China) dissolved in 0.5% sodium carboxymethylcellulose (China Jiaxing Sicheng Chemical Co., Ltd.)]. The probiotic cocktail (Shanghai Tongquan Biotechnology, Shanghai, China) contains *Bifidobacterium animalis* subsp. *lactis HN019, Bifidobacterium longum BI-05, Lactobacillus acidophilus NCFM, Lactobacillus rhamnosus Lr-32*, *Lactobacillus plantarum Lp-115*, *Lactobacillus salivarius Ls-33, Lactobacillus paracasei 37, Bifidobacterium animalis* spp. *lactis 420, Lactobacillus casei Lc-11*, and *Lactobacillus gasseri 36*, mixed in equal amounts. The total intervening amount was 8×10^10^ CFU per mouse per day for consecutive 7 days.

The animal protocols were approved by the Ethics Committee of Shanghai Tenth People’s Hospital affiliated to Tongji University (SHDSYY-2018-KY0008).

### FMT Preparation

(1) Twenty grams of fresh feces was collected from 25 healthy 6-week C57BL/6 mice using anal massage, 5 consecutive days before the experiment; (2) the feces were immediately mixed with 100 ml of sterile 10% glycerite and PBS mixture after the collection to get the feces mixture; (3) the fecal microbiota suspension was obtained by grinding the feces mixture with a standard mortar and pestle; (4) to remove the insoluble impurities, the fecal microbiota suspension was filtered through screens with the following diameters: 0.4 mm, 0.2 mm, and 0.1 mm; and (5) the suspension was collected and stored at −80°C (Wong et al., 2017).

### Assessment of Colonic Inflammation

Disease activity index (DAI) was used to assess the severity of colitis, including items of weight loss, stool consistency, and the presence of hematochezia, which were recorded every day during the experiment. DAI score calculation: (1) Bodyweight: 0 points were recorded if the bodyweight showed no decrease; 1 point was recorded when the bodyweight showed a 1%–5% decrease; 2 points were recorded when the bodyweight showed a 5%–10% decrease; 3 points were recorded when the bodyweight showed a 10%–15% decrease; and 4 points were recorded when the bodyweight showed more than 15% decrease. (2) Fecal traits: normal stool = 0 points; loose stool (not adhering to the anal paste of semi-formed stool) = 2 points; watery stool (can adhere to the anus watery stool) = 4 points. (3) Fecal occult blood result: no fecal occultation or naked eye blood stool = 0 points; fecal occult blood positive = 2 points; naked eye blood stool = 4 points. Finally, add the above three scores to get the DAI of each mouse to evaluate the severity of colitis (Tian et al., 2016; Li et al., 2019). The colon length and body weight alteration were also measured to evaluate the disease progression (Chen et al., 2020). We anesthetized mice with an inhalation anesthetic using 3% isoflurane during induction and 1.5% isoflurane during maintenance, then the serum was obtained by centrifuging blood using heart puncture drawn for 10 min at 3,000 rpm.

### Western Blot Analysis

Western blot assay in colon tissues was performed following the method reported previously (Kong et al., 2021b). Total protein from animal colon tissues was lysed with lysis buffer. The protein samples were loaded onto a 10% SDS–polyacrylamide gel and transferred to a polyvinylidene difluoride membrane (Simuwu-Biotechnology Co., Ltd, China, SD0043 or SD0044) before blocking with 5% skim milk powder for 4 h. After that, samples were incubated with the primary antibody overnight at 4°C: JAM-1 (Abcam, USA, ref#ab270446, 1:1,000), Occludin (Abcam, USA, ref#ab216327, 1:1,000), ZO-1 (Thermo Fisher Scientific, USA, ref#61-7300, 1:1,000), and β-Actin (Simuwu-Biotechnology Co., Ltd, China, ref#SD0034; 1:2,000). Next, the membrane was incubated with a horseradish peroxidase-conjugated secondary antibody (Simuwu-Biotechnology Co., Ltd, China, SD0038) for 1 h at room temperature. Immunodetection was performed using the Tanon 4600 chemiluminescence instrument (Tanon, Shanghai, China).

### Real-Time Quantitative PCR Analysis

Total RNA was extracted using Trizol reagent (Invitrogen, USA, ref#15596018). An RT reagent kit (TAKARA, Japan, ref#RR047A) was used to reverse-transcribe RNA into cDNA. A TB Green qPCR kit (TAKARA, Japan, ref#RR420A) was used to carry out the real-time qPCR reactions. The process was controlled and the result was analyzed on the qPCR instrument (Roche, LightCycler^®^ 480II). All the experiments were performed in triplicate and β-Actin was selected as the reference gene for mRNA. The specific primer sequences (Occludin, ZO-1, JAM-1, and β-Actin) used for amplification are listed in **Table 1**.

**Table 1:** Primer sequences used in this study.

Gene	Forward primer (5’–3’)	Reverse primer (5’–3’)
GAPDH-mouse NM_008084	AGGTCGGTGTGAACGGATTTG	TGTAGACCATGTAGTTGAGGTCA
JAM1-mouse NM_172647	TCTCTTCACGTCTATGATCCTGG	TTTGATGGACTCGTTCTCGGG
Occluding-mouse NM_008756	TTGAAAGTCCACCTCCTTACAGA	CCGGATAAAAAGAGTACGCTGG
ZO1-mouse NM_001163574	GCTTTAGCGAACAGAAGGAGC	TTCATTTTTCCGAGACTTCACCA

### Enzyme-Linked Immunosorbent Assay

The levels of cytokines (IL-4, IL-10, IL-17, IL-23, and IFN-γ) in the serum of mice were measured with the corresponding ELISA kit (Simuwu-Biotechnology Co., Ltd., ref#SDM0006 96T, ref#SDM0010 96T, ref#SDM0012 96T, ref#SDM0117 96T, and ref#SDM0115 96T). The whole process was carried out according to the protocol. (1) Dilute the animal serum 10 times with diluent and then add 100 μl to each well, mix the reaction plate, and leave the plate at 37°C for 40 min; (2) wash the reaction plate 3 times with washing liquid, and invert the plate on a filter paper to dry it; (3) add distilled water and 50 μl of the first antibody working liquid to each well (except blank), thoroughly mix the reaction plate, and place the plate at 37°C for 20 min; (4) wash the reaction plate 3 times with washing liquid as described before; (5) add 100 μl of enzyme-labeled antibody working solution to each well and place the reaction plate for 10 min at 37°C; (6) add 100 μl of the substrate working solution to each well and place the plate at 37°C in the dark for 15 min for reaction; (7) add 100 μl of the stop solution to each well and mix them well; (8) measure the absorbance value at 450 nm with a microplate reader (Tecan, F50) within 30 min.

### 16S rDNA Sequencing

The fecal samples were prepared according to the manufacturer’s instructions and the DNA was extracted from fecal samples as previously described (Kong et al., 2021a). A Nanodrop 2000 UV-vis spectrophotometer (Thermo Scientific, Wilmington, MA, USA) and 1% agarose gel electrophoresis were utilized to examine the concentrations and quality of the DNA samples. The specific forward primer 341F (5’-CCTACGGGRSGCAGCAG-3’) and reverse primer 806R (5’-GGACTACVVGGGTATCTAATC-3’) were designed to amplify the V3–V4 hypervariable regions of the bacteria 16S rDNA gene on a thermocycler PCR system. PCR products were detected by 2% agarose gel electrophoresis and were gelled and recovered by an AxyPrep DNA gel recovery kit (Axygen Biosciences, Union City, CA, USA). After quantification and homogenization, the DNA products underwent paired-end sequencing with Illumina NovaSeq PE250 (Illumina, San Diego, CA, USA). Statistical analysis was conducted in R (v3.5.1).

### Hematoxylin–Eosin Staining and Histopathology Evaluation

Paraffin-embedded colon tissues were cut into 5-mm sections. After being dewaxed in xylene and dehydrated in gradient alcohol, the sections were stained with hematoxylin for 8 min and with eosin for 5 min. Then, the sections were dehydrated and sealed. The histopathology evaluation was performed by two researchers who are blinded to section information. The following evaluation criteria were used: Epithelium (E): 0, normal morphology; 1, loss of goblet cells; 2, loss of goblet cells in large areas; 3, loss of crypts; 4, loss of crypts in large areas. Infiltration (I): 0, no infiltrate; 1, infiltrate around crypt basis; 2, infiltrate reaching to L. muscularis mucosae; 3, extensive infiltration reaching the L. muscularis mucosae and thickening of mucosa with abundant edema; 4, infiltration of the L. submucosa. The total histological score is defined as the sum of the epithelium and infiltration score (total score = E + I) (Obermeier et al., 1999).

### Metabolomics

The feces samples were prepared for metabolomic analysis according to the manufacturer’s instructions (Metabo-Profile Biotechnology, Shanghai). An ultra-performance liquid chromatography coupled to tandem mass spectrometry (UPLC-MS/MS) system (ACQUITY UPLC-Xevo TQ-S, Waters Corp., Milford, MA, USA) was used to quantitate the 7 main kinds of SCFAs (propanoic acid, isovaleric acid, isobutyric acid, butyric acid, valeric acid, hexanoic acid, and acetic acid) in this project as previously described (Liu Y et al., 2020). The raw data files generated by UPLC-MS/MS were processed using the MassLynx software (v4.1, Waters, Milford, MA, USA) to perform peak integration, calibration, and quantitation for each metabolite. Statistical analysis was conducted in Prism 8 (GraphPad) and R (v3.5.1).

### Statistical Analysis

All data were expressed as mean ± standard deviation (SD). The SPSS 22.0 software (SPSS Inc., Chicago, IL, USA) was utilized for statistical analyses. The Mann–Whitney *U* test and one-way ANOVA test were used to identify the significant differences between groups. Mann–Whitney *U* test and Pearson’s chi-square were utilized to identify the continuous and categorical variables, respectively. *p* < 0.05 was considered statistically significant.

## Results

### The Probiotic Cocktail Alleviates Intestinal Inflammation in DSS-Induced Colitis Mice

**Figure 1:**
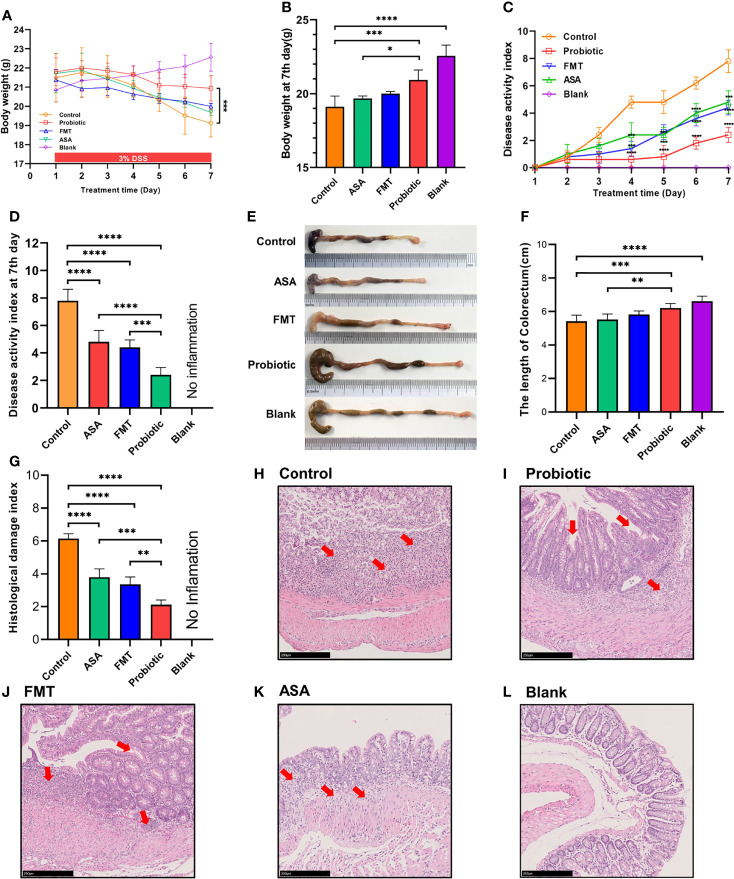
The probiotic cocktail alleviates intestinal inflammation in the DSS-induced colitis model. **(A)** Body weight of blank mice, and DSS-induced colitis mice treated with PBS, healthy mice donor FMT, 5-ASA, or the probiotic cocktail for 7 days. **(B)** The average body weight of each group at day 7. **(C)** The DAI score in each group. **(D)** The average DAI score at day 7. **(E)** Representative images of the colon in each group at day 7. **(F)** Colon length shown as a chart. **(G)** The HDI scores in each group based on inflammatory cell infiltration and mucosal damage under microscopic observation. **(H–L)** Representative image of colon tissue stained with H&E from each group. One-way ANOVA test was utilized, and data were shown as mean ± SD. *p < 0.05; **p < 0.01; ***p < 0.001; ****p < 0.0001. DSS, dextran sodium sulfate; PBS, phosphate buffered saline; FMT, fecal microbiota transplantation; 5-ASA, 5-aminosalicylic acid; DAI, disease activity index; HDI, histological damage index.

**Figure 2:**
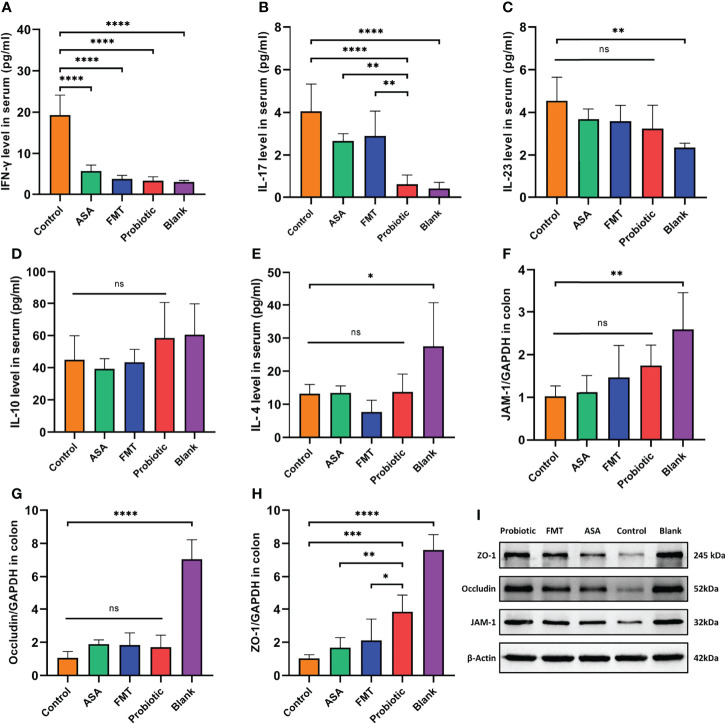
The probiotic cocktail repaired the gut barrier and reduced inflammatory factors in DSS-induced colitis. **(A–E)** The level of serum inflammatory cytokines (pro-inflammatory IFN-gamma, IL-17, and IL-23; anti-inflammatory IL-10 and IL-4) in blank mice, and DSS-induced colitis mice treated with PBS, healthy mice donor FMT, 5-ASA, or the probiotic cocktail. **(F–H)** The mRNA expression of tight junction protein (JAM-1, Occludin, ZO-1) of colon tissues in each group. **(I)** The protein expression of tight junction protein (JAM-1, Occludin, ZO-1) of colon tissue in each group. One-way ANOVA test was utilized, and data were shown as mean ± SD. *p < 0.05; **p < 0.01; ***p < 0.001; ****p < 0.0001; ns, no significant difference. DSS, dextran sodium sulfate; PBS, phosphate buffered saline; FMT, fecal microbiota transplantation; 5-ASA, 5-aminosalicylic acid.

As shown in **Figures 1A, B**, compared to the control group, the probiotic cocktail treatment significantly slowed down the weight loss of colitis mice, whereas the body weight among the FMT, 5-ASA, and control groups had no statistical difference. In evaluation of the severity of colitis, the DAI scores were found to significantly decrease in the probiotic cocktail, ASA, and FMT groups (**Figures 1C, D**), with the probiotic cocktail group obtaining the lowest score (**Figures 1C, D**). Furthermore, the probiotic cocktail notably increased the colon length of colitis mice compared to the control and ASA groups (**Figures 1E, F**). The images of representative hematoxylin–eosin (H&E) staining for colon and histological evaluation (**Figures 2G–L**) indicated that the probiotic cocktail performed best in alleviating the mucosal inflammation of DSS-induced colitis mice than any other treatment (red arrows represent enriched inflammatory cell and crypt for the probiotics group).

### The Probiotic Cocktail Reduces the Level of Serum Inflammatory Cytokines and Upregulates Tight Junction Proteins in DSS-Induced Colitis Mice

To further clarify the anti-inflammatory effect of the probiotic cocktail, we detected several inflammatory cytokines in the serum samples from colitis mice. As shown in **Figures 2A–E**, compared with the control group, the probiotic cocktail, FMT, and 5-ASA groups significantly decreased the level of pro-inflammatory IFN-γ and IL-17. The trend of decreased pro-inflammatory IL-23 and increased anti-inflammatory IL-10 and IL-4 was also observed in colitis mice after treatment of probiotic cocktail, although there was no significant difference (**Figures 2C–E**). Of note, the probiotic cocktail had a better inhibitory effect in serum IFN-γ and IL-17 production than FMT and 5-ASA (**Figures 2A, B**). Since our previous study revealed that probiotics played a crucial role in protecting the intestinal barrier (Liu et al., 2014; Yin et al., 2018), we also detected the expression of several tight junction proteins (JAM-1, ZO-1, and Occludin) in the colon tissues from colitis mice. As shown in **Figures 2F–H**, the probiotic cocktail significantly increased the mRNA expression of ZO-1 as compared with the control group, and the upward trend was also observed in JAM-1 and Occludin although not significant. Meanwhile, the following Western blot confirmed the upregulation of ZO-1, JAM-1, and Occludin at the protein level in the probiotic cocktail group (**Figure 2I**).

### The Probiotic Cocktail Improves the Gut Microbiota of DSS-Induced Colitis Mice

**Figure 3:**
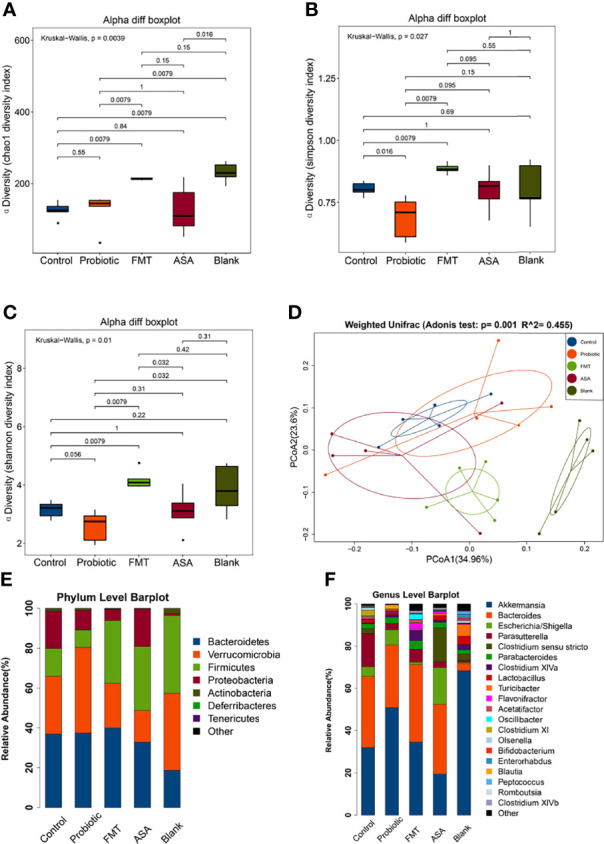
Diversity and composition of gut microbiota. **(A–C)** Alpha diversity was evaluated by the Chao1, Shannon, Simpson index among blank mice, and DSS-induced colitis mice treated with PBS, healthy mice donor FMT, 5-ASA, or the probiotic cocktail. **(D)** Weighted UniFrac PCoA analysis was utilized to distinguish bacterial clustering. The component proportion of the microbiome at the phylum **(E)** and genus level **(F)** among groups was shown. The relative abundance of genera difference was identified *via* the Wilcoxon rank-sum test. DSS, dextran sodium sulfate; PBS, phosphate buffered saline; FMT, fecal microbiota transplantation; 5-ASA, 5-aminosalicylic acid; PCoA, principal coordinate analysis.

To investigate the altered gut microbiota after different treatments, 16S rDNA sequencing was performed. Alpha diversity indexes were calculated to assess the differences in bacterial diversity among groups. Compared to the control group, the FMT group had a significantly higher Chao1, Shannon, and Simpson index (all *p* < 0.05), which was opposite for the probiotics group (**Figures 3A–C**). This finding suggested that the number and abundance of other species decreased after the probiotics were dominant. Meanwhile, the principal coordinate analysis based on weighted UniFrac distance revealed the significant differentiation in the composition of gut microbiota among groups (**Figure 3D**).

At the phylum level, the relative abundance of *Verrucomicrobia* was increased in the probiotics group but decreased in the FMT and 5-ASA groups (Control: 28.99%, Probiotics: 42.95%, FMT: 22.48%, ASA: 15.94%, Blank: 38.66%), while *Firmicutes* changed in an opposite manner (Control: 13.98%, Probiotics: 8.78%, FMT: 31.41%, ASA: 32.12%, Blank: 39.08%) (**Figure 3E**). In addition, the relative abundance of *Proteobacteria* was decreased in the probiotics and FMT groups as compared with that of the control group (Control: 18.66%, Probiotics: 9.78%, FMT: 5.69%, ASA: 18.85%, Blank: 0.91%). At the genus level, the relative abundance of *Akkermansia* and *Bifidobacterium* was increased in the probiotics group but decreased in the ASA group (Control: 31.98%, Probiotics: 50.84%, FMT: 34.58%, ASA: 19.40%, Blank: 68.37%; Control: 0.17%, Probiotics: 0.86%, FMT: 0.04%, ASA: 0.01%, Blank: 1.24%, respectively). Meanwhile, the relative abundance of *Parasutterella* was decreased in the probiotics, FMT, and ASA groups as compared with the control group (Control: 15.73%, Probiotics: 3.03%, FMT: 6.12%, ASA: 2.82%, Blank: 2.82%) (**Figure 3F**).

**Figure 4:**
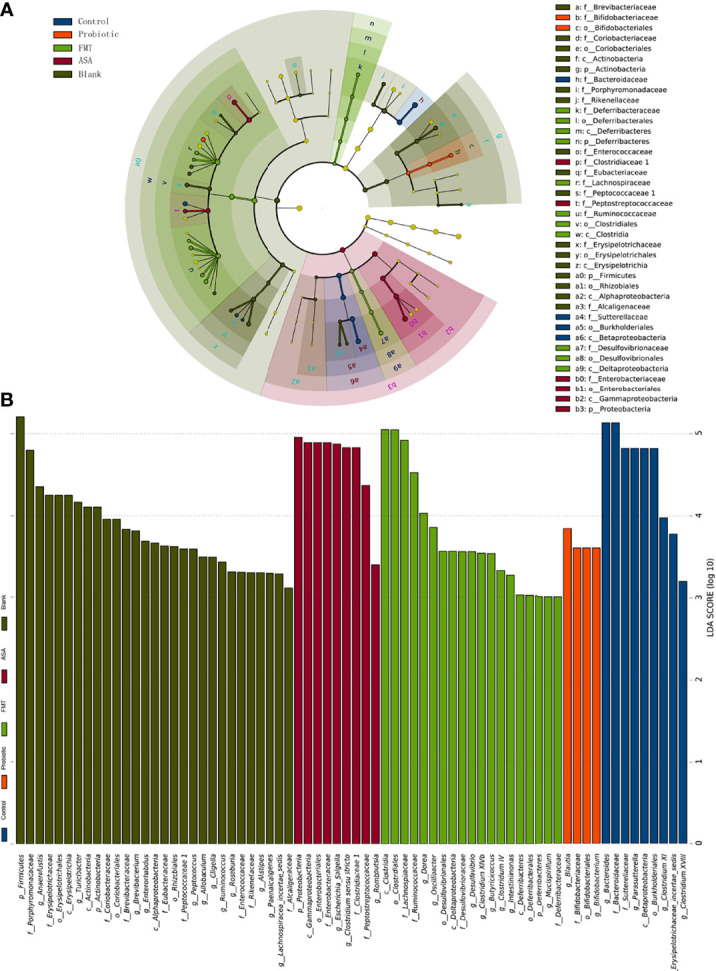
The result of linear discriminant analysis integrated with effect size (LEfSe). The phylogenetic distribution in blank mice, and DSS-induced colitis mice treated with PBS, healthy mice donor FMT, 5-ASA, or the probiotic cocktail was illustrated with a cladogram **(A)** and a histogram **(B)**. DSS, dextran sodium sulfate; PBS, phosphate buffered saline; FMT, fecal microbiota transplantation; 5-ASA, 5-aminosalicylic acid.

Furthermore, LEfSe analysis was utilized to figure out the key elements among different groups. At the genus level, DSS treatment significantly reduced the beneficial bacteria including *Bifidobacterium* and *Blautia*, which were reported to inhibit inflammation and enhance gut barrier (Chen et al., 2021). Notably, these two genera were obviously upregulated after the probiotic cocktail treatment. Moreover, we observed that the key genera in the FMT group were *Dorea, Oscillibacter, Desulfovibrio, Butyricicoccus, Micipirllum, Intestinimonas, Clostridium IV*, and *XIVb*, whereas the dominant bacteria in the ASA group were *Escherichia_Shigella*, *Clostridium sensu stricto*, and *Romboustia* (**Figures 4A, B**).

### The Probiotic Cocktail Promotes the Production of Beneficial Bacterial SCFAs

**Figure 5:**
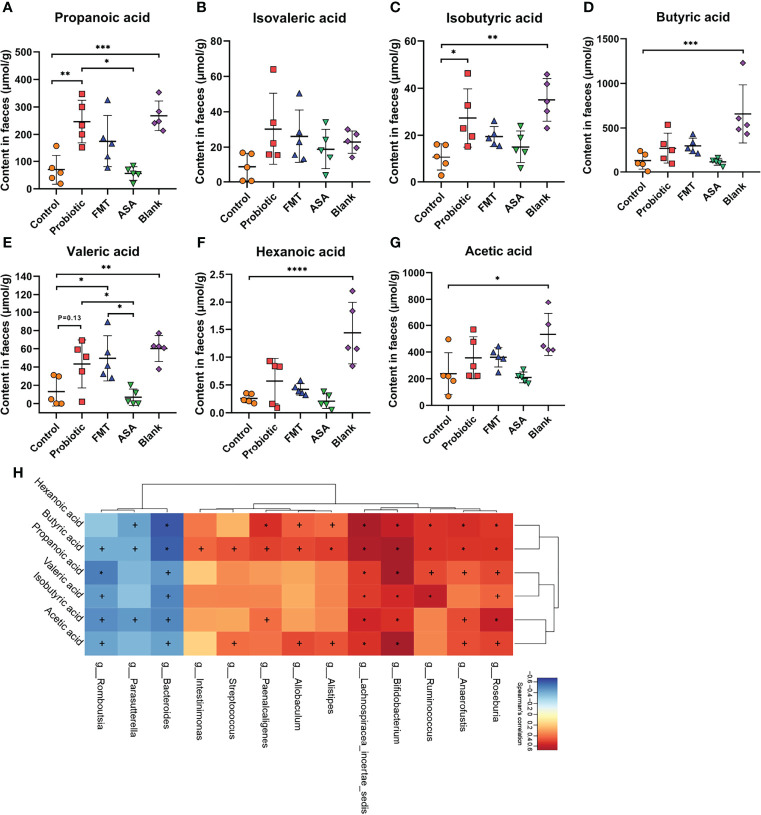
The concentration of fecal SCFAs and their correlation with the gut microbiome. **(A–G)** The concentration of 7 main kinds of SCFAs (propanoic acid, isovaleric acid, isobutyric acid, butyric acid, valeric acid, hexanoic acid, and acetic acid) in the feces of blank mice, and DSS-induced colitis mice treated with PBS, healthy mice donor FMT, 5-ASA, or the probiotic cocktail. One-way ANOVA test was utilized, and data were shown as mean ± SD. **(H)** The Spearman correlation analysis illustrated the relationship of SCFAs and gut microbiota. +*p* < 0.05; ∗*p* < 0.01. DSS, dextran sodium sulfate; PBS, phosphate buffered saline; FMT, fecal microbiota transplantation; 5-ASA, 5-aminosalicylic acid; SCFAs, short-chain fatty acids. *p < 0.05; **p < 0.01; ***p < 0.001; ****p < 0.0001.

Emerging evidence suggests that gut microbiota exerts an anti-inflammatory effect through producing SCFAs. Accordingly, we performed targeted metabolomics to detect the levels of several SCFAs (propionic acid, isovaleric acid, isobutyric acid, butyric acid, valeric acid, hexanoic acid, and acetic acid) in the fecal samples of DSS-induced colitis mice (**Figures 5A–G**). Compared with the control group, the probiotic cocktail rather than FMT and 5-ASA significantly promoted the production of propanoic acid and isobutyric acid (**Figures 5A, C**). The upward trend of isovaleric acid, butyric acid, valeric acid, hexanoic acid, and acetic acid was also observed in the probiotic cocktail group though not significant (**Figures 5B, D–G**). Additionally, Spearman rank correlation analysis was used to test correlations between bacteria and SCFA abundance (**Figure 5H**). Unsurprisingly, butyrate-producing bacteria *Bifidobacterium* and *Roseburia* were positively correlated with most SCFAs, while it was opposite for some pro-inflammatory bacteria such as *Parasutterella* and *Bacterioides*. Taken together, the results suggested that the probiotic cocktail effectively promoted the accumulation of both butyrate-producing probiotics and SCFAs.

## Discussion

**Figure 6:**
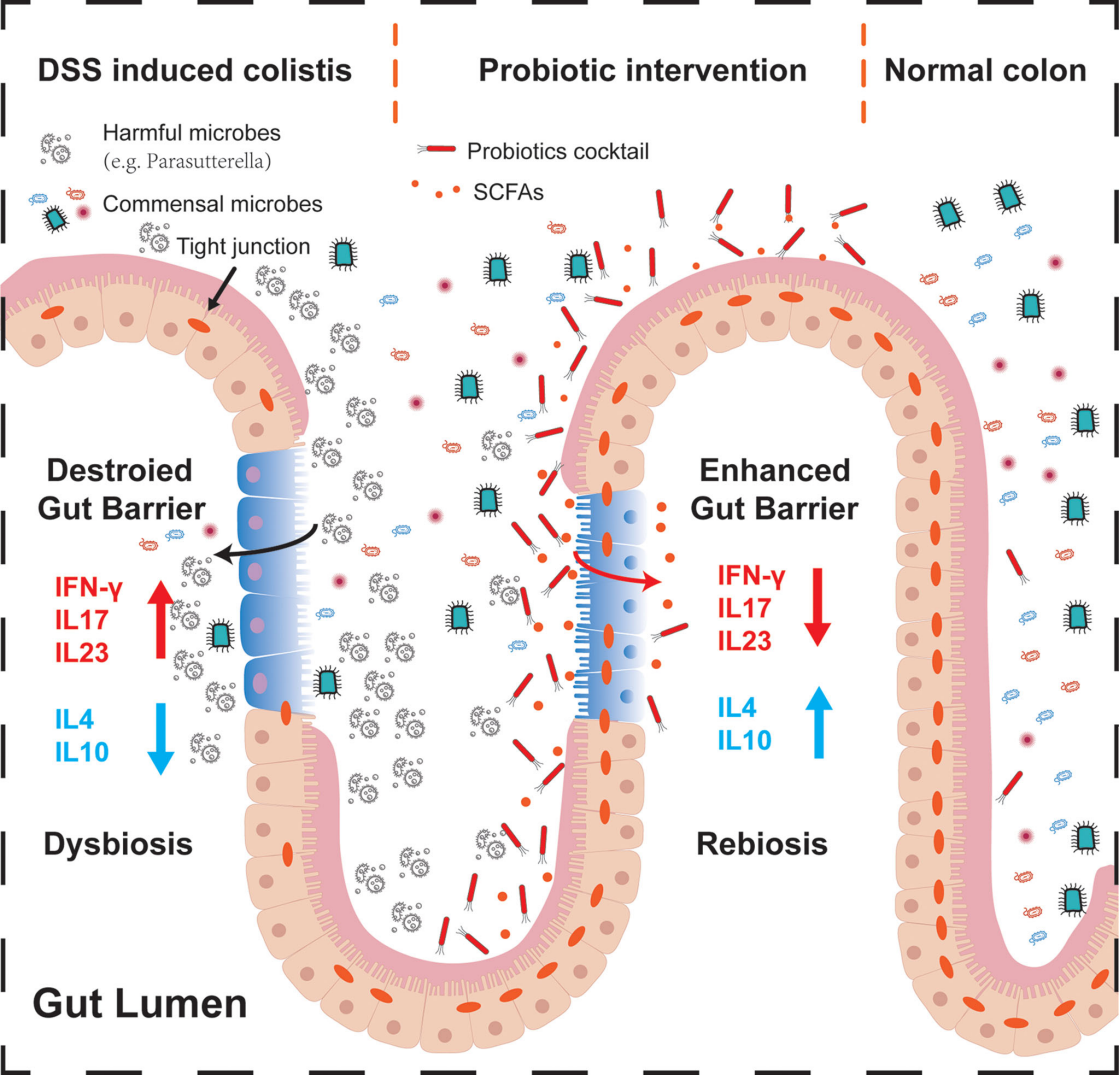
Schematic summary of the probiotic cocktail for alleviating acute intestinal inflammation. During the process of acute inflammation (left part), harmful bacteria may attach to the injured intestinal epithelium, inhibiting commensal microbes to colonize and make function, destroying the gut barrier and tight junction proteins by regulating pro- or anti-inflammatory factors. After probiotics intervention (middle part), colonized butyrate-producing probiotics *Bifidobacterium*, together with the associated SCFAs, alleviated the progression of acute intestinal inflammation through enhancing the intestinal barrier, regulating the muti-inflammatory factors, and expelling potential pathogenic bacteria. In the normal colon (right part), balanced commensal microbes interacted with the intestinal mucosa, contributing to the dynamic balance of host health.

In this study, we investigated the anti-inflammatory role of a novel probiotic cocktail in DSS-induced acute colitis mice. Remarkable differences are observed among healthy mice donor FMT, 5-ASA, and probiotics groups. Notably, probiotics treatment demonstrated its great value in preventing weight loss and intestinal contracture, upregulating SCFA-producing bacteria along with SCFA levels, and decreasing DAI score, pro-inflammatory factors, and intestinal barrier markers. These findings highlight that the probiotic cocktail could represent a promising dietary therapeutic strategy for IBD (**Figure 6**).

Exploring the mechanism revealed that probiotics intervention not only decreased the level of pro-inflammatory cytokines, namely, IFN-γ, IL-17, and IL-23, but also increased the anti-inflammatory cytokines IL-10 and IL-4, which may be mainly due to the successful colonization of both *Bifidobacterium* and *Lactobacillus*. Consistently, Bo et al. reported that *Bifidobacterium* significantly reduced IFN-γ and improved IL-10, alleviating enterotoxigenic *Escherichia coli*-induced diarrhea and can be a potential probiotic for clinical therapy (Yang et al., 2021). Yue et al. found that enterotoxigenic *E. coli*-induced diarrhea can also be suppressed by orally given *Lactobacillus plantarum via* regulating IFN-γ, IL-6, and IL-10; alternating gut microbes; and increasing gut SCFAs (Yue et al., 2020).

The intestinal barrier mainly consists of 3 kinds of barriers: (1) a physical barrier composed of tight epithelial junctions; (2) a secretory barrier composed of antimicrobial peptides and mucus; and (3) an immune barrier composed of immune cells or immune molecules (Camara-Lemarroy et al., 2018), which gets damaged under colitis conditions (Ungaro et al., 2017). Tight junction molecules are often used to evaluate intestinal barrier function in colitis (Furuse et al., 1993; Miner-Williams and Moughan, 2016; Mirsepasi-Lauridsen et al., 2019). Also, our results showed that probiotics increased the expression of intestinal mucosal tight junction proteins, represented by JAM-1, ZO-1, and Occludin, at both transcription and protein levels. These markers indicated the recovery of the gut barrier and the alleviation of colitis symptoms, which may be attributed to the effect of *Lactobacillus* and *Bifidobacterium*. As supported by a systematic review, *Lactobacillus* and *Bifidobacterium* exerted the anti-inflammation effect mainly through the enhancement of tight junction function in colorectal cancer patients (Wierzbicka et al., 2021). In addition, researchers found that the application of *Lactobacillus* and *Bifidobacterium* restored the gut barrier and defended gut-derived pathogenic infections in neonatal rats and dinitrobenzene sulfonic acid (DNBS)-induced colitis mice (Martín et al., 2016; Zeng et al., 2017).

Moreover, the microbiome and metabolome analysis indicated that probiotics treatment significantly improved the gut microbiota and promoted the production of bacteria-derived SCFAs. The microbiome composition displayed remarkable changes with the administration of the probiotic cocktail in this study, represented by increased *Akkermansia*, *Bifidobacterium*, and *Blautia*, and decreased *Parasutterella. Akkermansia* is a Gram-negative anaerobic bacterium that is reported to be selectively decreased in the fecal microbiome of IBD patients (Pittayanon et al., 2020). *Akkermansia* was also found to improve colitis-associated colorectal cancer and colitis through the alternation of cytotoxic T lymphocytes (CTLs), represented by CD8+ CTLs, CD16/32+ macrophages, and PD-1+ CTLs (Sun J et al., 2020). Moreover, our previous research showed that ketogenic diets improve colitis mice through enriching *Akkermansia*, enhancing intestinal barrier function, and reducing the production of group 3 innate lymphoid cells and associated inflammatory cytokines, which highlights the importance of our finding that *Akkermansia* was increased in the probiotics group (Kong et al., 2021a). Meanwhile, *Bifidobacterium* and *Blautia* were reported to alter gut microbiome dysbiosis, produce SCFAs, improve gut barrier function, block related pro-inflammatory cytokines, modulate T regulatory cells, and collectively blunt colitis in animal models (Kong et al., 2019; Sun S et al., 2020; Chen et al., 2021; Engevik et al., 2021; Song et al., 2021; Yang et al., 2021). However, *Parasutterella*, as a harmful bacterium that increased with the growth of age (Liu A et al., 2020), was found decreased after probiotics treatment. *Parasutterella* was also a key potentially pathogenic bacterium that is intensely associated with the progress of ulcerative colitis (Kim et al., 2020; Sun J et al., 2020). *Lactobacillus*, an SCFA-producing bacterium, was expected to increase but failed to populate during our research, probably due to its poor ability to colonize and the limited intervention time. However, its contribution can be seen through the alternation of *Akkermansia* and associated beneficial bacteria, which follows previous studies (Wang et al., 2021; Zhang et al., 2021).

Accumulating lines of evidence indicate that the intervention of probiotics makes it work through the alternation of fecal metabolites, especially SCFAs (Kong Q et al., 2021; Zhang et al., 2021). Thus, we performed the metabolome analysis using the fecal samples of colitis mice. Compared with the blank group, we found that DSS treatment significantly reduced the abundance of SCFAs, which could be reversed by the probiotics supplement, especially the upregulated propanoic acid and isobutyric acid, but not by healthy mice donor FMT and 5-ASA treatments. Clinical and basic studies confirm that microbiota-derived propionate acts directly on intestinal γδ T cells, inhibiting their production of IL-17 and IL-22 mainly through a histone deacetylase-dependent manner (Dupraz et al., 2021). Moreover, animal and human studies found that a similar increase in SCFAs protected liver function, reduced intestinal inflammation, and protected against colorectal cancer through regulating CRP level and hepatic lipid metabolism, decreasing proinflammatory Th1 and Th17 cells, and increasing anti-inflammatory immune cells (Alrafas et al., 2020; Ziętek et al., 2021). Furthermore, LeBlanc et al. claimed that *Bifidobacterium* produced acetate and formate in limited carbohydrate conditions, while supplying with carbohydrate could reproduce acetate and lactate (LeBlanc et al., 2017). Juneyoung et al. found that the transplantation of SCFA-producing bacteria represented by *Bifidobacterium* and *Lactobacillus* could alleviate neurological deficits and inflammation after stroke, and elevated SCFA concentrations of gut, brain, and plasma in aged stroke mice (Lee et al., 2020). In Spearman correlation analysis, beneficial bacteria including *Ruminococcus, Bifidobacterium*, and *Allobaculum* were significantly positively related to SCFA abundance, which was consistent with previous studies (Kong et al., 2019; Lee et al., 2020; Wang et al., 2020; Song et al., 2021). To sum up, the increased beneficial bacteria and their related metabolites (SCFAs) might be able to explain the mechanism by which probiotics alleviated the clinical and molecular changes associated with colitis.

These findings collectively supported the promising application of the probiotic cocktail in clinical practice. Despite our encouraging findings, there are several limitations that should be improved in our future work. Firstly, our work was only based on an animal model, and the actual therapeutic role of the probiotic cocktail in IBD patients remains undiscovered. This limitation is hoped to be solved by well-designed clinical trials based on sufficient patient resources. Secondly, the optimal working dose of the probiotic cocktail should be determined and more information on the affected microbiome should be provided by metagenomics sequencing instead of 16S rDNA sequencing. Finally, the molecular mechanisms of the probiotic cocktail in regulating barrier function and inflammatory cytokines still need to be further investigated in depth using *in vitro* and *in vivo* experiments.

## Conclusion

Our study for the first time demonstrated that a novel probiotic cocktail can effectively alleviate intestinal inflammation in DSS-induced colitis mice. Probiotic cocktail intervention significantly decreased the level of pro-inflammatory cytokines, upregulated the expression of tight junction proteins, improved gut microbiota, and promoted the production of beneficial SCFAs. Although clinical validations are necessary, the probiotic cocktail has great potential to be developed as an effective therapeutic strategy for IBD patients.

## Data Availability Statement

The data presented in the study are deposited in https://www.ncbi.nlm.nih.gov, accession number PRJNA819569.

## Ethics Statement

The animal study was reviewed and approved by the Ethics Committee of Shanghai Tenth People’s Hospital affiliated to Tongji University.

## Author Contributions

YFZ, YX, and XW contributed equally to this paper. YFZ and YX performed the experiments and drafted the manuscript. XW analyzed the data and helped with the polishing of the manuscript. LR and XY helped with the insightful discussions. YZ helped in attending to the mice. TS provided the probiotics and help in the gavage of mice. CK and LZ designed and supervised this study. All authors contributed to the article and approved the submitted version.

## Funding

This study was supported by a research grant from Shanghai Municipal Health and Family Planning Commission (201740234), the Probiotics International Research Institute of Xiuyisheng (JYJSKF-2018080001), the Program of Jiangsu Commission of Health (No. M2020024), the Social Development Program of Yangzhou Science and Technology Bureau (No. YZ2020078), and the Lijieshou Intestinal Barrier Foundation (LJS-201903B).

## Conflict of Interest

The authors declare that the research was conducted in the absence of any commercial or financial relationships that could be construed as a potential conflict of interest.

## Publisher’s Note

All claims expressed in this article are solely those of the authors and do not necessarily represent those of their affiliated organizations, or those of the publisher, the editors and the reviewers. Any product that may be evaluated in this article, or claim that may be made by its manufacturer, is not guaranteed or endorsed by the publisher.

## Abbreviations

DAI, disease activity index; DSS, dextran sulfate sodium; FMT, fecal microbiota transplantation; HDI, histological damage index; SCFAs, short-chain fatty acids; IBD, inflammatory bowel disease; 5-ASA, 5-aminosalicylic acid.

## References

[B1] Alrafas H. R. Busbee P. B. Chitrala K. N. Nagarkatti M. Nagarkatti P. (2020). Alterations in the Gut Microbiome and Suppression of Histone Deacetylases by Resveratrol Are Associated With Attenuation of Colonic Inflammation and Protection Against Colorectal Cancer. J. Clin. Med. 9 (6), 1796. doi: 10.3390/jcm9061796 PMC7355848 32526927

[B2] Burrello C. Garavaglia F. Cribiù F. M. Ercoli G. Lopez G. Troisi J.. (2018). Therapeutic Faecal Microbiota Transplantation Controls Intestinal Inflammation Through IL10 Secretion by Immune Cells. Nat. Commun. 9, 5184. doi: 10.1038/s41467-018-07359-8 30518790 PMC6281577

[B3] Camara-Lemarroy C. R. Metz L. Meddings J. B. Sharkey K. A. Yong V.W. (2018). The Intestinal Barrier in Multiple Sclerosis: Implications for Pathophysiology and Therapeutics. Brain: J. Neurol. 141, 1900–1916. doi: 10.1093/brain/awy131 PMC6022557 29860380

[B4] Chassaing B. Aitken J. D. Malleshappa M. Vijay-Kumar M. (2014). Dextran Sulfate Sodium (DSS)-Induced Colitis in Mice. Curr. Protoc. Immunol. 104, 15.25.1–15.25.14. doi: 10.1002/0471142735.im1525s104 24510619 PMC3980572

[B5] Chen Y. Yang B. Stanton C. Ross R. P. Zhao J. Zhang H.. (2021). Bifidobacterium Pseudocatenulatum Ameliorates DSS-Induced Colitis by Maintaining Intestinal Mechanical Barrier, Blocking Proinflammatory Cytokines, Inhibiting TLR4/NF-κb Signaling, and Altering Gut Microbiota. J. Agric. Food Chem. 69, 1496–1512. doi: 10.1021/acs.jafc.0c06329 33512996

[B6] Chen Y. Zhang L. Hong G. Huang C. Qian W. Bai T.. (2020). Probiotic Mixtures With Aerobic Constituent Promoted the Recovery of Multi-Barriers in DSS-Induced Chronic Colitis. Life Sci. 240, 117089. doi: 10.1016/j.lfs.2019.117089 31759038

[B7] Dias A. Douhard R. Hermetet F. Regimbeau M. Lopez T. E. Gonzalez D.. (2021). Lactobacillus Stress Protein GroEL Prevents Colonic Inflammation. J. Gastroenterol. 56, 442–455. doi: 10.1007/s00535-021-01774-3 33782752

[B8] Dupraz L. Magniez A. Rolhion N. Richard M. L. Da Costa G. Touch S.. (2021). Gut Microbiota-Derived Short-Chain Fatty Acids Regulate IL-17 Production by Mouse and Human Intestinal γδ T Cells. Cell Rep. 36, 109332. doi: 10.1016/j.celrep.2021.109332 34233192

[B9] Engevik M. A. Herrmann B. Ruan W. Engevik A. C. Engevik K. A. Ihekweazu F.. (2021). Bifidobacterium Dentium-Derived Y-Glutamylcysteine Suppresses ER-Mediated Goblet Cell Stress and Reduces TNBS-Driven Colonic Inflammation. Gut Microbes 13, 1–21. doi: 10.1080/19490976.2021.1902717 PMC8128206 33985416

[B10] Furuse M. Hirase T. Itoh M. Nagafuchi A. Yonemura S. Tsukita S.. (1993). Occludin: A Novel Integral Membrane Protein Localizing at Tight Junctions. J. Cell Biol. 123, 1777–1788. doi: 10.1083/jcb.123.6.1777 8276896 PMC2290891

[B11] Hernández-Chirlaque C. Aranda C. J. Ocón B. Capitán-Cañadas F. Ortega-González M. Carrero J. J.. (2016). Germ-Free and Antibiotic-Treated Mice are Highly Susceptible to Epithelial Injury in DSS Colitis. J. Crohns Colitis 10 (11), 1324–1335. doi: 10.1093/ecco-jcc/jjw096 27117829

[B12] Kerur B. Benchimol E. I. Fiedler K. Stahl M. Hyams J. Stephens M.. (2021). Natural History of Very Early Onset Inflammatory Bowel Disease in North America: A Retrospective Cohort Study. Inflamm. Bowel Dis. 27 (3), 295–302 doi: 10.1093/ibd/izaa080 32386060 PMC8177809

[B13] Kiesler P. Fuss I. J. Strober W. (2015). Experimental Models of Inflammatory Bowel Diseases. Cell Mol. Gastroenterol. Hepatol. 1 (2), 154–170. doi: 10.1016/j.jcmgh.2015.01.006 26000334 PMC4435576

[B14] Kim J. Choi J. H. Ko G. Jo H. Oh T. Ahn B.. (2020). Anti-Inflammatory Properties and Gut Microbiota Modulation of Porphyra Tenera Extracts in Dextran Sodium Sulfate-Induced Colitis in Mice. Antioxidants 9 (10), 988. doi: 10.3390/antiox9100988 PMC7602078 33066339

[B15] Kong C. Gao R. Yan X. Huang L. Qin H. (2019). Probiotics Improve Gut Microbiota Dysbiosis in Obese Mice Fed a High-Fat or High-Sucrose Diet. Nutr. (Burbank Los Angeles County Calif) 60, 175–184. doi: 10.1016/j.nut.2018.10.002 30611080

[B16] Kong Q. Wang B. Tian P. Li X. Zhao J. Zhang H.. (2021). Daily Intake of Lactobacillus Alleviates Autistic-Like Behaviors by Ameliorating the 5-Hydroxytryptamine Metabolic Disorder in VPA-Treated Rats During Weaning and Sexual Maturation. Food Funct. 12, 2591–2604. doi: 10.1039/D0FO02375B 33629689

[B17] Kong C. Yan X. Liu Y. Huang L. Zhu Y. He J.. (2021a). Ketogenic Diet Alleviates Colitis by Reduction of Colonic Group 3 Innate Lymphoid Cells Through Altering Gut Microbiome. Signal Transduct Target Ther. 6, 154. doi: 10.1038/s41392-021-00549-9 33888680 PMC8062677

[B18] Kong C. Yan X. Zhu Y. Zhu H. Luo Y. Liu P.. (2021b). Fusobacterium Nucleatum Promotes the Development of Colorectal Cancer by Activating a Cytochrome P450/epoxyoctadecenoic Acid Axis *via* TLR4/Keap1/NRF2 Signaling. Cancer Res. 81 (17), 4485–4498 doi: 10.1158/0008-5472.CAN-21-0453 34162680

[B19] LeBlanc J. G. Chain F. Martín R. Bermúdez-Humarán L. G. Courau S. Langella P. (2017). Beneficial Effects on Host Energy Metabolism of Short-Chain Fatty Acids and Vitamins Produced by Commensal and Probiotic Bacteria. Microbial Cell Fact 16, 79. doi: 10.1186/s12934-017-0691-z PMC5423028 28482838

[B20] Lee J. d'Aigle J. Atadja L. Quaicoe V. Honarpisheh P. Ganesh B. P.. (2020). Gut Microbiota-Derived Short-Chain Fatty Acids Promote Poststroke Recovery in Aged Mice. Circ. Res. 127, 453–465. doi: 10.1161/CIRCRESAHA.119.316448 32354259 PMC7415518

[B21] Li X. Tan J. Zhang F. Xue Q. Wang N. Cong X.. (2019). H.pylori Infection Alleviates Acute and Chronic Colitis With the Expansion of Regulatory B Cells in Mice. Inflammation 42, 1611–1621. doi: 10.1007/s10753-019-01022-0 31377948

[B22] Liu Y. Alookaran J. J. Rhoads J. M. (2018). Probiotics in Autoimmune and Inflammatory Disorders. Nutrients 10 (10), 1537. doi: 10.3390/nu10101537 PMC6213508 30340338

[B23] Liu W. Guo W. Hang N. Yang Y. Wu X. Shen Y.. (2016). MALT1 Inhibitors Prevent the Development of DSS-Induced Experimental Colitis in Mice *via* Inhibiting NF-κb and NLRP3 Inflammasome Activation. Oncotarget 7, 30536–30549. doi: 10.18632/oncotarget.8867 27105502 PMC5058699

[B24] Liu Z. Kang L. Li C. Tong C. Huang M. Zhang X.. (2014). Knockout of MIMP Protein in Lactobacillus Plantarum Lost its Regulation of Intestinal Permeability on NCM460 Epithelial Cells Through the Zonulin Pathway. BMC Gastroenterol. 14, 171. doi: 10.1186/1471-230X-14-171 25277875 PMC4287571

[B25] Liu Y. Kong C. Gong L. Zhang X. Zhu Y. Wang H.. (2020). The Association of Post-Stroke Cognitive Impairment and Gut Microbiota and its Corresponding Metabolites. J. Alzheimer’s Dis: JAD 73, 1455–1466. doi: 10.3233/JAD-191066 31929168

[B26] Liu A. Lv H. Wang H. Yang H. Li Y. Qian J. (2020). Aging Increases the Severity of Colitis and the Related Changes to the Gut Barrier and Gut Microbiota in Humans and Mice, The Journals of Gerontology. Ser. A Biol. Sci. Med. Sci. 75, 1284–1292. doi: 10.1093/gerona/glz263 32048723

[B27] Lloyd-Price J. Arze C. Ananthakrishnan A. N. Schirmer M. Avila-Pacheco J. Poon T. W.. (2019). Multi-Omics of the Gut Microbial Ecosystem in Inflammatory Bowel Diseases. Nature 569, 655–662. doi: 10.1038/s41586-019-1237-9 31142855 PMC6650278

[B28] Loddo I. Romano C. (2015). Inflammatory Bowel Disease: Genetics, Epigenetics, and Pathogenesis. Front. Immunol. 6, 551. doi: 10.3389/fimmu.2015.00551 26579126 PMC4629465

[B29] Magro F. Cordeiro G. Dias A. M. Estevinho M. M. (2020). Inflammatory Bowel Disease - Non-Biological Treatment. Pharmacol. Res. 160, 105075. doi: 10.1016/j.phrs.2020.105075 32653651

[B30] Martín R. Laval L. Chain F. Miquel S. Natividad J. Cherbuy C.. (2016). Bifidobacterium Animalis Ssp. Lactis CNCM-I2494 Restores Gut Barrier Permeability in Chronically Low-Grade Inflamed Mice. Front. Microbiol. 7, 608. doi: 10.3389/fmicb.2016.00608 27199937 PMC4858658

[B31] Miner-Williams W. M. Moughan P. J. (2016). Intestinal Barrier Dysfunction: Implications for Chronic Inflammatory Conditions of the Bowel. Nutr. Res. Rev. 29, 40–59. doi: 10.1017/S0954422416000019 27087106

[B32] Mirsepasi-Lauridsen H. C. Vallance B. A. Krogfelt K. A. Petersen A. M. (2019). Escherichia Coli Pathobionts Associated With Inflammatory Bowel Disease. Clin. Microbiol. Rev. 32 (2), e00060-18. doi: 10.1128/CMR.00060-18 30700431 PMC6431131

[B33] Obermeier F. Kojouharoff G. Hans W. Schölmerich J. Gross V. Falk W. (1999). Interferon-Gamma (IFN-Gamma)- and Tumour Necrosis Factor (TNF)-Induced Nitric Oxide as Toxic Effector Molecule in Chronic Dextran Sulphate Sodium (DSS)-Induced Colitis in Mice. Clin. Exp. Immunol. 116, 238–245. doi: 10.1046/j.1365-2249.1999.00878.x 10337013 PMC1905281

[B34] Oh N. S. Lee J. Y. Kim Y. T. Kim S. H. Lee J. H. (2020). Cancer-Protective Effect of a Synbiotic Combination Between Lactobacillus Gasseri 505 and a Cudrania Tricuspidata Leaf Extract on Colitis-Associated Colorectal Cancer. Gut Microbes 12, 1785803. doi: 10.1080/19490976.2020.1785803 32663105 PMC7524312

[B35] Okayasu I. Hatakeyama S. Yamada M. Ohkusa T. Inagaki Y. Nakaya R. (1990). A Novel Method in the Induction of Reliable Experimental Acute and Chronic Ulcerative Colitis in Mice. Gastroenterology 98 (3), 694–702. doi: 10.1016/0016-5085(90)90290-H 1688816

[B36] Pittayanon R. Lau J. T. Leontiadis G. I. Tse F. Yuan Y. Surette M.. (2020). Differences in Gut Microbiota in Patients With vs Without Inflammatory Bowel Diseases: A Systematic Review. Gastroenterology 158, 930–946.e1. doi: 10.1053/j.gastro.2019.11.294 31812509

[B37] Seishima J. Iida N. Kitamura K. Yutani M. Wang Z. Seki A.. (2019). Gut-Derived Enterococcus Faecium From Ulcerative Colitis Patients Promotes Colitis in a Genetically Susceptible Mouse Host. Genome Biol. 20, 252. doi: 10.1186/s13059-019-1879-9 31767028 PMC6876129

[B38] Song W. Sun L. Y. Zhu Z. J. Wei L. Qu W. Zeng Z. G.. (2021). Characteristics of Gut Microbiota in Children With Biliary Atresia After Liver Transplantation. Front. Physiol. 12, 704313. doi: 10.3389/fphys.2021.704313 34262484 PMC8273867

[B39] Stojanov S. Berlec A. Štrukelj B. (2020). The Influence of Probiotics on the Firmicutes/Bacteroidetes Ratio in the Treatment of Obesity and Inflammatory Bowel Disease. Microorganisms 8 (11), 1715. doi: 10.3390/microorganisms8111715 PMC7692443 33139627

[B40] Sun J. Chen H. Kan J. Gou Y. Liu J. Zhang X.. (2020). Anti-Inflammatory Properties and Gut Microbiota Modulation of an Alkali-Soluble Polysaccharide From Purple Sweet Potato in DSS-Induced Colitis Mice. Int. J. Biol. Microb. 153, 708–722. doi: 10.1016/j.ijbiomac.2020.03.053 32169445

[B41] Sun S. Luo L. Liang W. Yin Q. Guo J. Rush A. M.. (2020). Bifidobacterium Alters the Gut Microbiota and Modulates the Functional Metabolism of T Regulatory Cells in the Context of Immune Checkpoint Blockade. Proc. Natl. Acad. Sci. U. S. A. 117, 27509–27515. doi: 10.1073/pnas.1921223117 33077598 PMC7959554

[B42] Thia K. T. Loftus E. V. Jr. Sandborn W. J. Yang S. K. (2008). An Update on the Epidemiology of Inflammatory Bowel Disease in Asia. Am. J. Gastroenterol. 103, 3167–3182. doi: 10.1111/j.1572-0241.2008.02158.x 19086963

[B43] Tian Z. Liu J. Liao M. Li W. Zou J. Han X.. (2016). Beneficial Effects of Fecal Microbiota Transplantation on Ulcerative Colitis in Mice. Dig Dis. Sci. 61, 2262–2271. doi: 10.1007/s10620-016-4060-2 26846120

[B44] Ungaro R. Mehandru S. Allen P. B. Peyrin-Biroulet L. Colombel J. F. (2017). Ulcerative Colitis. Lancet (Lond. Engl.) 389, 1756–1770. doi: 10.1016/S0140-6736(16)32126-2 PMC6487890 27914657

[B45] Wang P. Wang J. Li D. Ke W. Chen F. Hu X. (2020). Targeting the Gut Microbiota With Resveratrol: A Demonstration of Novel Evidence for the Management of Hepatic Steatosis. J. Nutr. Biochem. 81, 108363. doi: 10.1016/j.jnutbio.2020.108363 32388250

[B46] Wang J. Zhang J. Liu W. Zhang H. Sun Z. (2021). Metagenomic and Metatranscriptomic Profiling of Lactobacillus Casei Zhang in the Human Gut. NPJ Biofilms Microb 7, 55. doi: 10.1038/s41522-021-00227-2 PMC8249650 34210980

[B47] Wierzbicka A. Mańkowska-Wierzbicka D. Mardas M. Stelmach-Mardas M. (2021). Role of Probiotics in Modulating Human Gut Microbiota Populations and Activities in Patients With Colorectal Cancer-A Systematic Review of Clinical Trials. Nutrients 13 (4), 1160. doi: 10.3390/nu13041160 33915854 PMC8066620

[B48] Wijmenga C. (2005). Expressing the Differences Between Crohn Disease and Ulcerative Colitis. PloS Med. 2, e230. doi: 10.1371/journal.pmed.0020230 16120009 PMC1196472

[B49] Wong S. H. Zhao L. Zhang X. Nakatsu G. Han J. Xu W.. (2017). Gavage of Fecal Samples From Patients With Colorectal Cancer Promotes Intestinal Carcinogenesis in Germ-Free and Conventional Mice. Gastroenterology 153, 1621–1633.e6. doi: 10.1053/j.gastro.2017.08.022 28823860

[B50] Yang B. Huang Z. He Z. Yue Y. Zhou Y. Ross R. P.. (2021). Protective Effect of Bifidobacterium Bifidum FSDJN7O5 and Bifidobacterium Breve FHNFQ23M3 on Diarrhea Caused by Enterotoxigenic Escherichia Coli. Food Funct. 12 (16), 7271–7282. doi: 10.1039/D1FO00504A 34165468

[B51] Yin M. Yan X. Weng W. Yang Y. Gao R. Liu M.. (2018). Micro Integral Membrane Protein (MIMP), a Newly Discovered Anti-Inflammatory Protein of Lactobacillus Plantarum, Enhances the Gut Barrier and Modulates Microbiota and Inflammatory Cytokines. Cell. Physiol. Biochem: Int. J. Exp. Cell. Physiol Biochem Pharmacol. 45, 474–490. doi: 10.1159/000487027 29402771

[B52] Yue Y. He Z. Zhou Y. Ross R. P. Stanton C. Zhao J.. (2020). Lactobacillus Plantarum Relieves Diarrhea Caused by Enterotoxin-Producing Escherichia Coli Through Inflammation Modulation and Gut Microbiota Regulation. Food Funct. 11, 10362–10374. doi: 10.1039/D0FO02670K 33220669

[B53] Zeng Q. He X. Puthiyakunnon S. Xiao H. Gong Z. Boddu S.. (2017). Probiotic Mixture Golden Bifido Prevents Neonatal Escherichia Coli K1 Translocation *via* Enhancing Intestinal Defense. Front. Microbiol. 8, 1798. doi: 10.3389/fmicb.2017.01798 28979247 PMC5611410

[B54] Zhang Q. Fan X. Y. Cao Y. J. Zheng T. T. Cheng W. J. Chen L. J.. (2021). The Beneficial Effects of Lactobacillus Brevis FZU0713-Fermented Laminaria Japonica on Lipid Metabolism and Intestinal Microbiota in Hyperlipidemic Rats Fed With a High-Fat Diet. Food Funct. 12 (16), 7145–7160 doi: 10.1039/D1FO00218J 34231612

[B55] Ziętek M. Celewicz Z. Kikut J. Szczuko M. (2021). Implications of SCFAs on the Parameters of the Lipid and Hepatic Profile in Pregnant Women. Nutrients 13 (6), 1749. doi: 10.3390/nu13061749 34063900 PMC8224042

